# The Effect of Physical Activity on Bone Biomarkers in People With Osteoporosis: A Systematic Review

**DOI:** 10.3389/fendo.2020.585689

**Published:** 2020-10-23

**Authors:** Sofia Marini, Giuseppe Barone, Alice Masini, Laura Dallolio, Laura Bragonzoni, Yari Longobucco, Francesca Maffei

**Affiliations:** ^1^Department of Life Quality Studies, University of Bologna, Campus of Rimini, Rimini, Italy; ^2^Department of Biomedical and Neuromotor Science, University of Bologna, Bologna, Italy; ^3^Clinical and Experimental Medicine Unit, Department of Medicine and Surgery, University of Parma, Parma, Italy

**Keywords:** physical activity, exercise, training, bone biomarkers, osteoporosis, bone metabolism, bone turnover

## Abstract

**Background:**

Bone imbalance between anabolic and catabolic processes at the level of remodeling unit due to the prevalence of resorbing activity, represents a health problem of aging. The consequence is the negative balance of bone turnover that can lead to osteoporosis. Physical activity (PA) can play a central role in the comprehensive management of osteoporosis, since it induces the anabolism of bone tissue. Bone turnover biomarkers, reflecting the cellular activity linked to bone metabolism, can represent an evaluation tool to assess the efficacy of PA in the osteoporotic population. The aim of this systematic review, conducted according to the Preferred Reporting Items for Systematic Reviews and Meta-Analyses (PRISMA) statement, was to investigate the effects of PA interventions on bone biomarkers in people with osteoporosis.

**Methods:**

A comprehensive literature search of electronic databases was conducted through PubMed, Cochrane, Cinahl, Embase, Trip, to find randomized controlled trials (RCTs) investigating the topic of PA and bone turnover biomarkers in the osteoporosis population. In accordance with the Cochrane risk-of-bias tool, the quality of each study was assessed.

**Results:**

Out of 992 identified articles, 136 full texts were screened. Only three RTCs matched the eligibility criteria. In one study, sub-maximal aerobic exercise improved Bone-specific alkaline phosphatase (bone formation biomarker) and Amino-terminal Crosslinked Telopeptide of type 1 collagen (bone resorption biomarker) in osteoporotic women. The other two studies showed a positive effect on total alkaline phosphatase (a non-specific bone formation biomarker) in women with osteoporosis.

**Conclusion:**

The systematic review revealed possible exercise benefits in terms of improving bone formation and decreasing bone resorption biomarkers in the osteoporotic population. However, these results should be interpreted with caution, especially due to the limited number and poor quality of the studies included. Further research is needed to estimate the influence of PA on bone biomarkers in the osteoporosis management.

## Introduction

Bone is hard tissue that is in a constant state of flux, being built up by bone-forming cells called osteoblasts while also being broken down or resorbed by cells known as osteoclasts (1). The assessment of bone quality can involve several parameters, including the extent of mineralization, the number and distribution of micro fractures, the rate of osteocyte apoptosis, and changes in the collagenous bone matrix. The status of bone mass is usually measured using a densitometry method (2). However, it is more difficult to accurately examine bone structure and strength in live tissue only by Dual X-ray Absorptiometry (DXA) (3). Some blood and urinary molecules have been identified as biomarkers to detect the dynamics of bone turn-over (4). They are ideal tools to evaluate the actual metabolic status of the bone, as well as a well-established result of abnormal metabolism (5).

**Table 1** shows the most reviewed bone biomarkers to assess the different phases of bone metabolism process (4, 6–11).

**Table 1:** Summary of bone turnover biomarkers currently available and their characteristics.

Biomarkers	Assay method	Characteristics	Reference
**Bone formation markers**			
Alkaline phosphatase (ALP)	Serum Standard Auto-analyzer technique	Widely used but non-specific for bone turnover	Vasikaran et al. (6); Kuo and Chen (4)
Bone alkaline phosphatase (BALP)	Serum EIA-CLEIA	Applied for the monitoring of osteoporosis.	Kuo and Chen (4); Nagy et al. (7); Park et al. (8)
Osteocalcin (OC)	Serum IRMA-ECLIA	No significant utility for the assessment of osteoporosis. Promising for the investigation of osteoporosis therapy efficacy	Liu et al. (9); Kuo and Chen (4); Nagy et al. (7)
Procollagen type 1 C-terminal propeptide (P1CP)	Serum Radioimmunoassay	Limited study in literature. Promising for the investigation of bone formation	Kuo and Chen (4); Nagy et al. (7)
Procollagen type 1 N-terminal propeptide (P1NP)	Serum RIA-ECLIA	The most sensitive marker to measure the bone formation rate the most accepted marker for monitoring drug therapy.	Kuo and Chen (4); Glendenning et al. (10); Nagy et al. (7)
**Bone resorption markers**			
Amino-terminal crosslinked telopeptide of type 1 collagen (NTX-1)	Urine EIA-CLEIA	Stable and not affected by food intake. Promising marker for osteoporosis management	Kuo and Chen (4); Nagy et al. (7)
Bone sialoprotein (BSP)	Serum immunoassay	Potential biomarker for osteoporosis assessment	Kuo and Chen (4)
Carboxy-terminal crosslinked telopeptide of type 1 collagen (CTX-1)	Serum/plasma/urine EIA-CLEIA	Specific and sensitive biomarker for osteoporosis management. Useful marker for monitoring drug therapy	Kuo and Chen (4); Glendenning et al. (10); Nagy et al. (7)
Cathepsin K (CTSK)	Serum ELISA	Potential marker for monitoring drug therapy	Kuo and Chen (4); Drake et al. (11)
Deoxypyridinoline (DPD)	Urine HPLC-EIA-CLEIA	Not very sensitive for osteoporosis management	Kuo and Chen (4); Nagy et al. (7)
Hydroxylysine (HYL)	Urine HPLC	Limited application due to the lack of a simple routine method	Kuo and Chen (4)
Hydroxyproline (HYP)	Urine Spectrophotometric technique	Not very sensitive for osteoporosis management, it has been replaced by more specific markers	Kuo and Chen (4)
Osteopontin (OP)	Plasma ELISA	Promising biomarker to monitor the parathyroid hormone treatment in menopausal osteoporosis	Kuo and Chen (4); Glendenning et al. (10); Nagy et al. (7)
Pyridinoline (PYD)	Urine HPLC	Non-specific for diagnosis and treatment of osteoporosis	Kuo and Chen (4); Nagy et al. (7)
Tartrate-resistant acid phosphatase 5b (TRAP 5b)	Serum/plasma EIA	Good specificity and high sensitivity for monitoring drug therapy	Kuo and Chen (4); Nagy et al. (7)
**Regulators of bone turnover**			
Dickkopf-1 (DDK-1)	Serum ELISA	Insufficient clinical data for osteoporosis management	Kuo and Chen (4); Nagy et al. (7)
Osteoprotegerin (OPG),	Serum ELISA	Insufficient data for clinical management of osteoporosis	Kuo and Chen (4)
Receptor activator of NF-kB ligand (RANKL)	Serum ELISA	Insufficient data for diagnosis and treatment of osteoporosis	Kuo and Chen (4)
Sclerostin	Serum ELISA	Insufficient clinical data for osteoporosis assessment	Kuo and Chen (4); Nagy et al. (7)

The negative balance of bone turnover, due to the absolute (increase in osteoclastic function) or relative (inadequacy of osteoblastic function) prevalence, represents a health problem. The most common cause of this process is aging, but it can also result from other conditions such as immobilization, cortisone therapy, or estrogen deficiency. The most common metabolic bone disease is osteoporosis, which is characterized by low bone mass and structural deterioration of bone tissue, leading to bone fragility and an increased susceptibility to fractures (12). Currently, it has been estimated that more than 200 million people are suffering from osteoporosis, and this number is increasing due to the aging population and the change in lifestyles. According to recent statistics from the International Osteoporosis Foundation, osteoporosis affects one in three women and one in five men over the age of 50 years worldwide (13). Estrogen deficiency is the main etiopathogenic factor in postmenopausal osteoporosis. Indeed, throughout the menopausal transition, serum estradiol and estrone levels decrease, with an increase in bone resorption leading to osteoporosis (14, 15).

Nowadays, osteoporosis is a major public health concern worldwide due to its healthcare cost and requires a multi-modal care approach including both pharmacological and physical activity interventions (16). In Europe, the most commonly administered agents involved in osteoporosis drug therapy are raloxifene, bisphosphonates, agents derived from parathyroid hormone, and denosumab (17, 18). The guidelines for the prevention and treatment of osteoporosis recommend regular physical exercise. A low level of physical activity (PA) represents an important risk factor for osteoporosis due to the reduced mechanical stimulation of osteoblasts. For these reasons, PA should be part of the comprehensive management of osteoporotic patients since it can reduce disability, improve physical function, lower the risk of subsequent falls, and act on bone structure (19, 20).

It is likely that PA induces an anabolic or homeostatic effect on bone *via* mechanotransduction (21). Although the mechanism underlying the effects of exercise on bone remodeling is not yet fully understood, some hypotheses seem more probable. One is the piezoelectric effect: when the mechanical impulse transmitted to the bone is converted by hydroxyapatite crystals into an electrical impulse that leads to greater bone mineralization. Another is the vascular effect: when the increase in muscle activity leads to a positive variation in the bone blood flow, improving the local metabolism (22). In particular, exercise carried out under conditions of weight-bearing determines the most significant benefits, as the mechanical stress is more intense. Also, the bone response to exercise is greater in districts where more mechanical stress is exerted. Furthermore, aerobic exercise seems to be particularly effective in the enzymatic activation of the osteoblasts (23).

Nowadays bone metabolic biomarkers have become useful clinical parameters in the management of osteoporosis and their use continues to expand (24), as the possible variation in their concentrations may indicate an anabolism status or a bone catabolism (25). The monitoring of bone turnover biomarkers could be a useful assessment tool to understand the physiological mechanism deriving from the osteogenic effect of PA (26) and to assess the impact of exercise on osteoporotic bone (27, 28). This highlights the need for an investigation of the influence of exercise on biomarkers linked to bone turnover in the osteoporotic population. In this scenario, the purpose of the present systematic review was to evaluate and critically analyze, for the first time, the available evidence on the effects of PA interventions on bone biomarkers in people with osteoporosis.

## Methods

### Search Strategy and Data Sources

We conducted this current Systematic Review following the Preferred Reporting Items for Systematic Reviews and Meta-analyses (PRISMA) guidelines (29). Beforehand, we registered the protocol in the International Prospective Register of Systematic Reviews (PROSPERO).

The following PICO (Patients, Interventions, Comparators and Outcomes) question was developed, addressing the primary search objective, through the following search terms: (P) Osteoporotic people, aged 45–80+; (I) Physical activity; (C) Standard care or no exercise treatment; (O) The effect of physical activity interventions on bone biomarkers.

We searched electronic databases, with a 10-year time limit on the publication date because we were interested in recent pharmacologic treatments and approaches. The primary search was performed on 20 October 2019 and was updated on 14 May 2020. In all data bases we applied the following criteria to define the research: we included only Clinical Trial, Clinical Study, Comparative Study, Observational Study, Randomized Controlled Trial with Full text available, published in the last 10 years; with Human subjects. We defined a range of population aged 80 and over: 80+ years, Middle Aged + Aged: 45+ years, Middle Aged: 45–64 years, Aged: 65+ years.

The databases searched were: MEDLINE (PubMed); Embase (Ovid); Cochrane Central Register of Controlled Trials (Central); CINAHL (EBSCO); TRIP Medical. The search terms were adapted when necessary to fit the specific search requirements of each database.

Search strategies (strings adapted to the different databases) used the following Boolean expression: keywords and terms: “(((((((((((((((((((((Osteoporoses) OR Osteoporosis, Post-Traumatic) OR Osteoporosis, Post Traumatic) OR Post-Traumatic Osteoporoses) OR Post-Traumatic Osteoporosis) OR Osteoporosis, Senile) OR Osteoporoses, Senile) OR Senile Osteoporoses) OR Osteoporosis, Involutional) OR Senile Osteoporosis) OR Osteoporosis, Age-Related) OR Osteoporosis, Age Related) OR Bone Loss, Age-Related) OR Age-Related Bone Loss) OR Age-Related Bone Losses) OR Bone Loss, Age Related) OR Bone Losses, Age-Related) OR Age-Related Osteoporosis) OR Age Related Osteoporosis) OR Age-Related Osteoporoses) OR Osteoporoses, Age-Related) AND (((((((((((((((((((((((((Exercises) OR Physical Activity) OR Activities, Physical) OR Activity, Physical) OR Physical Activities) OR Exercise, Physical) OR Exercises, Physical) OR Physical Exercise) OR Physical Exercises) OR Acute Exercise) OR Acute Exercises) OR Exercise, Acute) OR Exercises, Acute) OR Exercise, Isometric) OR Exercises, Isometric) OR Isometric Exercises) OR Isometric Exercise) OR Exercise, Aerobic) OR Aerobic Exercise) OR Aerobic Exercises) OR Exercises, Aerobic) OR Exercise Training) OR Exercise Trainings) OR Training, Exercise) OR Trainings, Exercise) AND ((((((((((((((((((Bones and Bone Tissue) OR Bones and Bone) OR Bone Tissue) OR Bone Tissues) OR Tissue, Bone) OR Tissues, Bone) OR Bony Apophyses) OR Apophyses, Bony) OR Bony Apophysis) OR Apophysis, Bony) OR Condyle) OR Condyles) OR Bones) OR Bone) OR Bone Biomarker) OR Bone Biomarkers) OR Biomarker, Bone) OR Biomarkers, Bone)”. After exporting articles, duplicates were removed. Moreover, we conducted a gray literature search of other papers and hand searches of key conference proceedings, journals, professional organizations’ websites and guideline clearing houses. In accordance with the snowball technique, we examined references cited in the primary papers to identify additional papers.

### Inclusion and Exclusion Criteria

Inclusion criteria:

1. Articles written in English;
2. Population with a diagnosis of osteoporosis (T score ≤−2.5);
3. Physical activity intervention;
4. Bone Biomarker evaluation, bone biomarkers measured at least one time during the study;
5. Additional physical performance measured outcomes, or other indices of physical performance described in each study for example walking, balance, dexterity;
6. All the additional outcomes measured at least one time during the study;
7. Original primary data.

Exclusion criteria:

1. Articles not pertinent for the research topic;
2. Population with osteopenia, absence of osteoporosis diagnosis, different diseases;
3. Absence of physical activity intervention, physiotherapy, reported physical activity, other therapy;
4. Study protocol or other papers without original data.

### Data Extraction and Quality Assessment

Four independent and blind investigators (SM, AM, GB, YL) screened and checked all the titles and abstracts retrieved in order to select pertinent items and to extract data following the inclusion criteria, using a pre-tested data extraction form. In case of doubts about the pertinence, the investigators assessed the eligibility of the study by reading the full text of the article.

The studies thus selected were independently and blindly assessed for the risk of bias by three researchers (SM, AM, GB), using the “Cochrane risk-of-bias tool for randomized trials” (30). Any disagreement between the quality scores separately assigned by the blind reviewers was resolved through discussion and, if necessary, a fourth blind reviewer (YL) was involved as tiebreaker. The evaluation of risk of bias was made on the basis of the primary outcome of our interest, namely bone turnover biomarkers. This methodological choice was supported by the PRISMA guidelines (29).

The Cochrane risk-of-bias tool for randomized trials analyses seven bias categories for studies classified as a randomized controlled trial (RCT): (1) random sequence generation and (2) allocation concealment (concerning bias of selection and allocation), (3) selective reporting for reporting bias, (4) blinding of participants and personnel (performance bias due to knowledge of the allocated intervention), (5) blinding of outcome assessment for detection bias, (6) incomplete outcomes data for bias in attrition, and another domain (7) called “other bias” based on the probable bias not covered in the other categories. Each category results in a value of high, low or unclear (when the authors did not provide enough evidence about the bias category) risk of bias. According to the Cochrane RoB Tool we converted the score to AHRQ (Agency for Healthcare Research and Quality) standards (Good, Fair and Poor). The threshold to provide the final score are the following: Good quality correspond to all criteria met (*i.e.* low risk of bias for each domain); Fair quality, only one criterion not met (*i.e.* high risk of bias for one domain) or two criteria unclear; Poor quality two or more criteria listed as high or unclear risk of bias.

The investigators extracted data independently, following the standardized norms for literature collection. We conducted a descriptive analysis of the studies by searching and extracting the following information from the articles: name of the first author, publication year, country, study design, population study with ages and number of experimental (EG) and control (CG) groups, sample size, type intensity and frequency of intervention, primary and secondary outcomes, results stratifying the studies for the different outcomes. Results were tabulated as mean ± SD where possible.

Any disagreement was resolved by consensus (LD, LB, FM). The study authors or investigators were contacted when additional information was necessary (31).

## Results

### Study Selection and Characteristics

**Figure 1:**
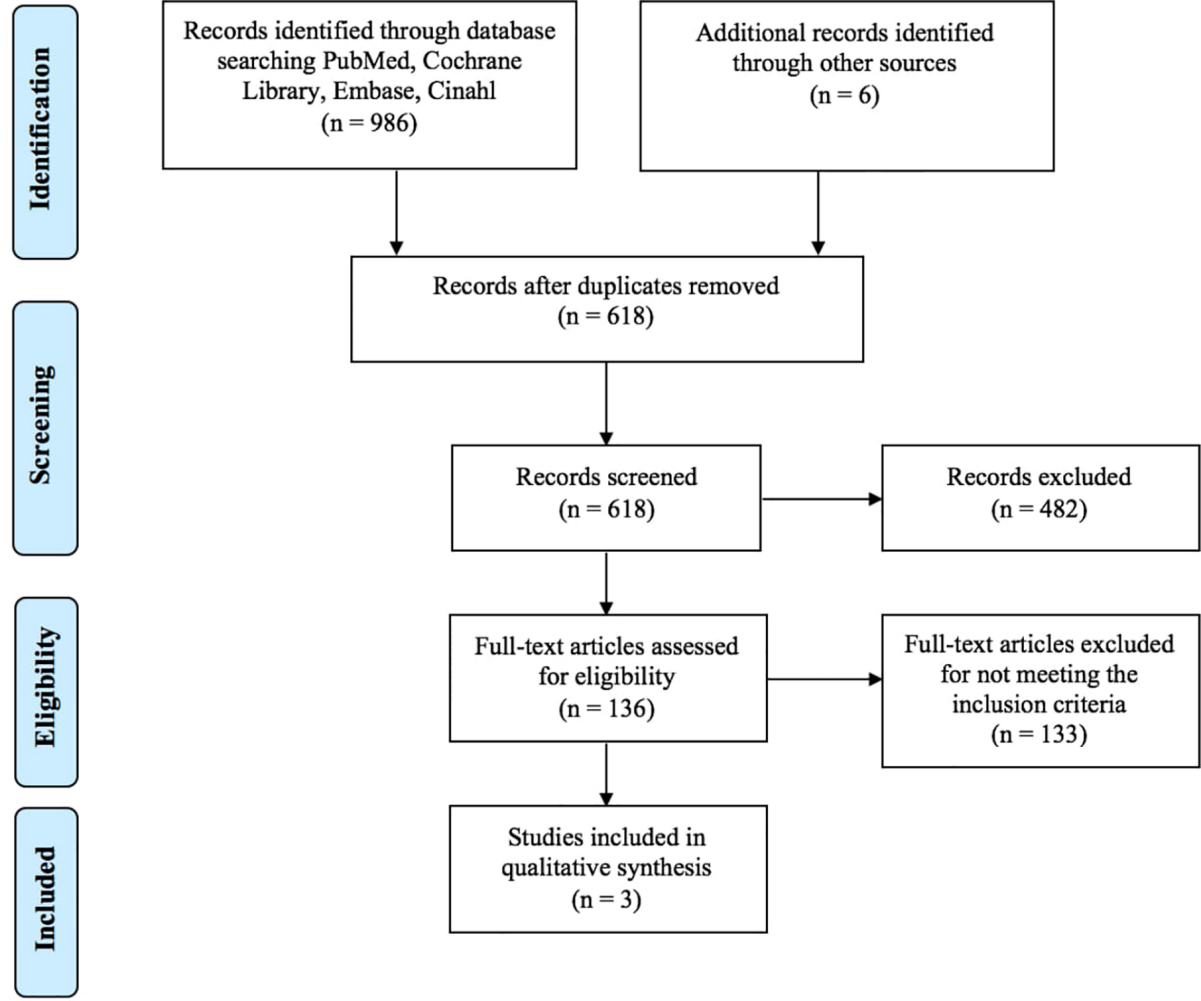
PRISMA diagram of the study selection.

As shown in **Figure 1**, a total of 992 articles were identified in the databases browsed and through hand search. Papers were published from 2012 to 2018; 374 studies were excluded because duplicated, 482 studies were excluded following abstract and/or title review. After this step, we judged 136 records as pertinent, 133 of which were subsequently excluded after a detailed full-text reading. The main causes of exclusion were related to the non-relevance and coherence with the aim of this study: the effects of PA interventions on bone biomarkers in people with osteoporosis. Furthermore, the majority of the articles were excluded due to the samples that did not match our inclusion criteria (people with osteopenia and not osteoporosis). As a result, only three papers (32–34) were finally included in the systematic review, fully meeting the eligibility criteria (**Figure 1**).

### Risk of Bias

Following the descriptive analysis, we assessed the quality of each RCT study.

In accordance with the Cochrane risk-of-bias tool for randomized trials we assessed the quality based on biomarkers outcome (see **Supplementary Material, Figure S1**). The three RCTs do not explain in detail the randomization methods or allocation of participants (items #1 and #2), and none of them had a research protocol registered; due to this, the selective reporting was assessed as unclear (item#3).

There was no blinding of participants (item #4), but the review authors judge that the biomarker outcome is not likely to be influenced by lack of blinding of participants. Regarding the blinding of outcome assessment (item #5) Roghani et al. was the only one that described and used techniques and methods that ensure the sensitivity of outcome assessment (32); the studies by Arazi et al. (33) and El-Mekawy et al. (34) were not clear in describing the methodology used to guarantee no risk of bias of outcome assessors. Overall, each RCT had one or more criteria unclear. For these reasons, the risk of bias was scored as “Poor quality”.

### Data Extraction

According to our aim focused on assessing the effects of PA on biomarkers, we extracted the data considering the bone biomarkers analysis and other hematological parameters as primary outcome; bone mineral density (BMD) assessment and physical performance tests as secondary outcome. **Table 2** shows the main characteristics and results of the included studies evaluating the effects of PA interventions on bone biomarkers, in people with osteoporosis. The geographic origin of the studies was: Iran (n = 2, 66%) and Egypt. Study characteristics were heterogeneous. The sample size varied from 26 to 60 people. Ages ranged from 30–45 to 60–65 years. Concerning the subject’s inclusion/exclusion criteria, in both Roghani et al. and El-Mekawy et al. studies, subjects were excluded if they were taking any drugs that affected bone metabolism or were receiving hormone replacement therapy. In Arazi et al. an inclusion criterion was not using low-fat dairy (milk, yogurt, cheese) as a source of vitamin D. The duration of the intervention varied from 6–10 weeks to 6 months with a common frequency of three times a week. The type of exercise training was, in all three studies (26–28), aerobic such as walking on a treadmill or resistance weighted exercise, administered in more than one group. In both Roghani et al. and Arazi et al. were enrolled other experimental groups performing weighted aerobic exercise and aerobic-resistance training, respectively, while only the study by El-Mekawy included an outdoor walking intervention. The El-Mekawy et al. study did not have a control group, while the other two envisaged a standard care control group. In Arazi et al. two intervention groups (concurrent training and milk; only milk supplementation) received a supplementation of 500 ml daily milk for ten weeks.

**Table 2:** Studies included in the review.

Study	Study design	Sample	Intervention	Outcomes	Results	Quality (RoB Tool)
Arazi et al. (33) Rasht, Iran	RCT	N:40 age:30–45 EG-training:10 EG-training + milk:10 EG-milk:10 CG:10	**Duration:** 10 weeks **Type of intervention:** EG-training: aerobic exercises 10 weeks × three sessions/week, 90–110 min × session; EG-training + milk: aerobic exercises 10 weeks × three sessions/week, 90–110 min × session + 500 ml daily milk for 10 weeks immediately (250 ml) and 1 hour after training (250 ml). EG-milk: 500 ml daily milk for 10 weeks, milk immediately (250 ml) and 1 h after training (250 ml), CG: standard care.	**Primary outcome**: ALP and 25OHD **Secondary outcome**: BMD hip values (right and left) and BMD lumbar spine (L2–L4)	**Primary outcome results** **Statistically significant improvement in ALP:** EG-training + milk p < 0.001; EG-training p < 0.001; EG-milk p = 0.01. **Statistically significant improvement in 25OHD:** EG-training + milk p<0.001; EG-training p < 0.001; EG-milk p = 0.03. **Secondary outcome results** **Statistically significant improvement in BMD hip** EG-training+milk: right hip: p < 0.001; left hip: p < 0.001; EG-training: right hip: p = 0.01; left hip: p < 0.001; EG-milk: right hip: p = 0.15; left hip: p = 0.09. **Statistically significant improvement in BMD lumbar spine** EG-training + milk p = 0.02; EG-training p < 0.001; EG-milk p = 0.10.	**Poor**
El-Mekawy et al. (34) Cairo, Egypt	RCT	N: 60 womenage:59.03 ± 2.67 EG-A:20 EG-B:20 EG-C:20	**Duration:** 6 months **Frequency:** 3 times a week **Type of intervention:** EG-A: walk daily in the morning in fresh air, 30 min. EG-B: aerobic exercise training for hip and lumbar spine. Sustained muscle contraction for each specific exercise was maintained for 5 s followed by 10 s of relaxation. EG-C: treadmill program for 30 min consisted of 5 min warm-up which involved walking with no resistance and no inclination at the walk way of the treadmill followed by 20 min of walking with 15° inclination at the walk way of the treadmill at 60–75% of the training heart rate and ended by 5 min cool down.	**Primary outcome**: ALP and calcium (Ca) **Secondary outcome**: Response of BMD neck and BMD lumbar spine to exercise	**Primary outcome results** **Pre–post change in ALP**: EG-A = pre: 175.68 ± 33.48 *vs* post: 173.00 ± 32.95, change pre:1.53%, p value < 0.91; EG-B = pre: 157.00 ± 35.23 *vs* post: 154.44 ± 35.92, change: 1.63%, p value < 0.33; EG-C = pre: 153.48 ± 36.44 *vs* post: 150.96 ± 35.92, change: 1.64%, p value < 0.05. **Pre–post change in Ca**: EG-A= pre: 8.48 ± 0.31 vs post: 8.66 ± 0.3, change: 2.12%, p-value<0.81; EG-B= pre: 8.45 ± 0.36 vs post: 8.66 ± 0.37, change: 2.49%, p value<0.44; EG-C= pre: 8.48 ± 0.34 vs post: 8.73 ± 0.37, change: 2.95%, p-value<0.66. **Secondary outcome results** **Pre–post change in BMD neck**: EG-A= pre: -2.97± 0.64 vs post: -2.66 ± 0.59, change:10.44%, p value<0.05; EG-B = pre: −2.87 ± 0.67 *vs* post: -2.55 ± 0.65, change: 11.15%, p-value < 0.004; EG-C = pre: 2.71 ± 0.30 *vs* post: −2.38 ± 0.32, change: 12.18%, p value < 0.002. **Pre–post change in BMD lumbar spine**: EG-A = pre: −3.59 ± 0.90 *vs* post: −3.26 ± 0.88, change: 9.19%, p-value < 0.01; EG-B = pre: −3.64 ± 0.65 *vs* post: −3.29 ± 0.74, change: 9.62%, p value < 0.002; EG-C = pre: −3.44 ± 0.83 *vs* post: −3.08 ± 0.79, change: 10.47%, p-value < 0.001.	**Poor**
Roghani et al. (32) Thran, Iran	RCT	N:27 age:45–65 CG:9 EG-aerobic:9 EG-weight:9	**Duration:** 6 weeks, 18 sessions **Frequency:** 3 times a week, 30 min each session **Type of intervention:** EG-Aerobic: treadmill submaximal, increasing the intensity every 2 weeks; EG-Weighted: Aerobic + wearing a vest 4–8% of body weight; CG: usual care	**Primary outcome**: (tALP), BALP, and NTX levels, calcium (Ca), phosphorus (P). **Secondary outcome**: Balance: near tandem stand (NTS) and star-excursion (SE) test	**Primary outcome results** **Pre–Post change in tALP (U/L)**: EG-Aerobic **=** pre: 218.00 ± 68.32 *vs* post: 226.12 ± 72.11, change: +8.12, NS; EG-Weighted **=** pre: 222.44 ± 60.96 *vs* post: 221.55 ± 80.04, change: −0.89, NS; CG = pre: 181.50 ± 83.36 *vs* post: 186.70 ± 80.04, change: +5.2, NS. **Pre–Post change in BALP (U/L)**: EG-Aerobic = pre: 156.12 ± 38.08 *vs* post: 173.37 ± 51.20, change: +10.25%, p = 0.03; EG-Weighted= pre: 154.22 ± 33.73 *vs* post: 166.44 ± 43.92, change: +7.31%, p = 0.05; CG = pre: 139.70 ± 59.55 *vs* post: 136.60 ± 57.37, change: −1.93%. **Pre–Post change in NTX (nM)**: EG-Aerobic = pre: 20.80 ± 2.37 *vs* post:19.51 ± 1.88, change: −5.99%, p = 0.001; EG-Weighted **=** pre: 21.10 ± 2.33 *vs* post: 19.72 ± 1.91, change: −6.34%, p = 0.002; CG = pre: 21.08 ± 2.32 *vs* post: 21.20 ± 2.38, change: +0.60%, p = 0.6. **Pre–Post change in P(mg/dl):** EG-Aerobic = pre: 3.86 ± 0.40 *vs* post: 3.84 ± 0.3, change: −0.02, NS; EG-Weighted = pre: 3.33 ± 0.43 *vs* post: 3.53 ± 0.26, change: +0.2, NS; CG = pre: 3.79 ± 0.42 *vs* post: 3.83 ± 0.66, change: +0.4, NS. **Pre–Post change in Ca (mg/dl)**: EG-Aerobic = pre: 9.10 ± 0.11 *vs* post: 9.16 ± 0.25, change: +0.06, NS; EG-Weighted **=** pre: 8.91 ± 0.16 *vs* post: 9.23 ± 0.23, change: +0.32, p-value < 0.07; CG = pre: 9.06 ± 0.38 *vs* post: 9.07 ± 0.20, change: +0.01, NS. **Secondary Outcome Results** **Pre–Post change in SE test (cm):** EG-Aerobic = +10.72%, p-value < 0.05; EG-Weighted= +13.43%, p-value < 0.05; CG = −10.43%, p-value < 0.05. **Pre–Post change in Near Tandem Stand (NTS) test (s)** EG-Aerobic **=** +49.68%, EG-Weighted= +104.66%, CG **=** −28.96%.	**Poor**

In the study by Arazi et al. the aim was to investigate the effects of concurrent training and milk, only training and daily milk consumption, on bone biomarkers and BMD. The exercise protocol for the concurrent training was performed by groups in 10 weeks, with three sessions of 90–110 min each week. Aerobic training included three sets of 5 min, running with 55–75% of heart rate maximum (HRmax) of the target and exercise intensity gradually increased for 5% HRmax and 3–5 min every two weeks (rest period of approximately 3 min between each set). Resistance training involved performing two sets of bench press, leg extension, wide grip pull-down, and leg curls, which were circular with 10 RM, and training intensity was gradually increased every two weeks for new 10 RM. At the end of 10 weeks, Arazi et al. reported a significant improvement in blood levels of 25-hydroxyvitamin D (25OHD) and ALP in all the experimental groups (concurrent training-milk, training group, milk group) compared to the control group (standard care), with a higher increase in the concurrent training-milk group (p < 0.05).

The study by El-Mekawy et al. conducted to determine the ideal type of exercise for the treatment of osteoporosis, foresaw three types of exercise (brisk walking in fresh air, specific exercise program for hip and lumbar spine, and weight-bearing exercise program on treadmill). The results obtained after 6 months showed a significant increase in all the primary and secondary tested parameters (pre-post change in ALP, BMD neck and BMD lumbar spine) in the three exercise groups.

Roghani et al. evaluated the effect of submaximal aerobic exercise with and without external loading, in three groups: aerobic group, weighted-vest group and control group. The exercise program performed by both the aerobic and weighted-vest group, consisted of 18 sessions of submaximal aerobic walking exercise on a treadmill three times a week, every other day, with each session lasting 30 min. The intensity of the exercise was increased gradually during the 6 weeks; specifically, 50% heart rate reserve (HRR) during the first 2 weeks, 55% HRR during the second 2 weeks, and 60% HRR during the last 2 weeks. Heart rate, blood pressure (BP), and electrocardiogram (ECG) were monitored throughout the course of the exercise program. In the weighted-vest group the initial inner weight of the vest was 4% of the individual’s body weight and was gradually increased by 2% every 2 weeks based on the tolerance level of each subject. The control group was requested not to change their daily physical activity or dietary patterns during the 6 weeks. As a result, BALP and NTX decreased significantly in both exercise groups (p < 0.05). The changes in bone biomarker levels were significant between each exercise group compared to the control group, except for the ALP pre-post changes. Concerning the secondary outcome of balance assessed through the near tandem stand (NTS) and star-excursion (SE) test, the exercise groups increased significantly while the control group decreased (p < 0.05).

## Discussion

The present systematic review evaluates the effects of PA on bone biomarkers in the osteoporotic population and provides an outlook of their application to set up exercise programs.

Most of the articles included in the preliminary full text analysis from the database research involved osteopenic people without osteoporosis, and they did not meet the established inclusion criteria. For this reason, our findings focused on data from only three studies (32–34). Regarding the bone biomarkers assessment, all the studies investigated the serum ALP. Roghani et al. and El-Mekawy et al. both included serum calcium as an additional parameter, while Arazi et al. analyzed the 25-hydroxyvitamin D (25OHD).

All three studies included in our review reported a significant improvement in terms of bone biomarkers value in osteoporotic people participating in exercise interventions. The best effect in bone turnover was obtained with two different PA interventions including both aerobic and weighted-vest aerobic training in the study by Roghani et al. In particular, the study showed that short term submaximal walking training wearing a weighted vest is effective for stimulating bone formation and decreasing bone resorption in postmenopausal women with osteoporosis.

According to more recent literature (4, 7, 10) the most specific and sensitive biomarkers for osteoporosis management and the most accepted for monitoring drug therapy are CTX-1(bone resorption) and P1NP (bone formation). These two biomarkers were not investigated in any of the three studies analyzed. Roghani et al. evaluated BALP, a widely-used bone formation biomarker, and NTX, a promising marker of bone resorption. On the other hand, both El-Mekawy et al. and Arazi et al. investigated ALP, a non-specific bone turnover marker, even though widely applied in clinical diagnosis. These data hamper a robust evaluation of the findings.

Regarding quality assessment, the studies analyzed present further limitations due to the low quality. All three RCTs were scored as “Poor quality” according to the Cochrane Tool for Quality Assessment. In all the included studies, it was not possible to understand the methodology used for randomization and allocation concealment. Moreover, the three studies did not register the study protocol. Only Roghani et al. described specific methods to guarantee the sensitivity of outcomes assessment. Despite these limitations, Roghani et al. could be considered the most appropriate study with a lesser number of risks of bias.

As already mentioned in the *Introduction* of this article, osteoporosis has been increasingly studied over the years as it is a skeletal disease leading to structural deterioration of bone tissue and especially when related fractures occur, it significantly interferes with the quality of life (35–37). Besides, concern has grown to identify effective strategies for managing osteoporosis.

Evidence has consistently proven the importance of regular participation in specific exercise programs to prevent and minimize the osteoporotic bone deterioration and its consequences on health (19, 20, 38). In this review bone mineral density assessment and physical performance tests have been evaluated as secondary outcome. Roghani et al. showed that weighed-vest aerobic exercise is more effective for improving the balance of participants than simple aerobic training. The other two studies evaluated the effect of PA on BMD estimated with DXA. El-Mekawy et al. reported an increase in BMD at neck and lumbar spine with the highest score for the weight bearing exercise group, and the lowest recorded in the brisk walking group. Arazi et al. showed that the concurrent training-milk intervention significantly improved the BMD measured at lumbar spine and hips.

To date, no optimal exercise training for osteoporotic people has been established, but there is growing evidence supporting a multimodal approach that includes different types of exercise and training (39, 40). Resistance training and weight-bearing impact exercises seem to be the most suitable and specific to reduce the risk of fracture, acting on the musculoskeletal system; however, the benefits depend on the frequency and intensity of training (40). Balance and mobility exercises are also widely used to increase functionality and reduce the risk of falls (41). On the other hand, aerobic PA that does not include impact (*e.g.* cycling or swimming) has a weak effect on prevention related to bone loss, due to the low impact on the musculoskeletal apparatus, inadequate to gain a bone adaptation (42). In spite of this, aerobic exercises have great benefits on the cardiovascular and metabolic apparatus and body composition of osteoporotic patients. In addition, exercise can help to achieve beneficial and significant effects on quality of life, balance, and functional mobility also in patients with osteoporosis-related vertebral fractures (43, 44). However, there is still no agreement on which type of exercise, in terms of intensity, frequency, duration, type and setting, is optimal and can affect bone metabolism in people with osteoporosis (45–47).

Biomarkers of bone metabolism, reflecting the cellular activity linked to the bone turnover process, could be a valid tool to assess the efficacy of PA and exercise programs in the osteoporotic population. Of note, some studies, which we excluded after our preliminary full-test analysis because they include non-osteoporotic study groups, monitored the benefits of physical activity on bone metabolism by the evaluation of P1NP and CTX, the two biomarkers considered specific for bone turnover (44, 48). Interestingly, an improvement in bone metabolism was induced by different types of exercise, for example a football training intervention (49, 50). Moreover, Moreira et al. found a positive effect of high-intensity aquatic exercise on P1NP and CTX among people with osteoporosis and osteopenia on P1NP and CTX (51). On the other hand, Wochna et al. did not obtain effects on CTX in healthy post-menopausal women performing aqua fitness activities in deep water (52).

On the whole, the available scientific evidence points to a gap of knowledge regarding the potential of PA to influence biomarkers and does not allow an unequivocal conclusion about exercise programs suitable for people with osteoporosis. Despite the limitations reported in terms of the small sample size of the studies included and their quality and design, to our knowledge this systematic review is the first that investigates the effects of PA on bone biomarkers in the osteoporotic population. Hopefully, our findings can serve to summarize the existing literature on this topic and highlight the need for additional studies in this field.

Further research is required with a special focus on osteoporotic people, investigating the most specific bone biomarkers (CTX, P1NP) and following the guidelines on quality evidence to adopt more rigorous methodologies. In the future, bone turnover biomarkers could prove highly promising in the design and evaluation of exercise programs for osteoporosis interventions.

## Conclusion

For the understanding of the physical activity role in osteoporosis management, a desired goal is to correlate the effects of exercise on bone turn-over biomarkers. Despite our comprehensive literature search, the level of available evidence does not allow us to establish a clear conclusion since the limit number of the studies and their poor quality according to Risk of Bias tool.

Although the results should be interpreted with caution, the reported data indicate the beneficial effect of exercise especially weighted and aerobic, in terms of improving bone formation biomarkers such as ALP and BALP, and decreasing bone resorption biomarkers such as NTX in the osteoporotic population. These findings could pave the way for planning future research to better assess the effectiveness of PA on bone metabolism. Further study population, performed with rigorous methodology, is needed to identify the most useful exercise able to modulate bone turnover biomarkers in people with osteoporosis.

## Data Availability Statement

The original contributions presented in the study are included in the article/**Supplementary Material**. Further inquiries can be directed to the corresponding author.

## Author Contributions

SM, FM, and LD conceived and designed the systematic review. SM, AM, GB, and YL independently reviewed abstracts and papers and disagreements were resolved by consensus with FM. SM, AM, GB, and YL acquired, analyzed, and interpreted the data. FM checked data extractions. SM drafted the manuscript, which was critically revised for important intellectual content by all authors. AM wrote sections of the manuscript, GB and YL revised the manuscript and contributed with intellectual ideas. FM, LD, and LB supervised the study. All authors contributed to the article and approved the submitted version.

## Conflict of Interest

The authors declare that the research was conducted in the absence of any commercial or financial relationships that could be construed as a potential conflict of interest.

## Supplementary Material

Supplementary Figure 1 Risk of Bias evaluation (**Supplementary Material**).
