# Supplementary material for: The Effect of Physical Activity on Bone Biomarkers in People With Osteoporosis: A Systematic Review
Source: Front Endocrinol (Lausanne). 2020 Oct 23;11:585689. doi: 10.3389/fendo.2020.585689 (PMC7644859; doi:10.3389/fendo.2020.585689)
Supplement: Supplementary Figure 1 — Risk of Bias evaluation (Supplementary Material). [file Image_1.pdf]

Figure S1. Risk of Bias evaluation

Studies	Random sequence	Allocation concealment	Selective reporting	Other bias	Blinding of participants	Blinding of outcome assessment	Incomplete outcome data	Quality
Arazi et al. 2018 (27)								Poor
El-Mekawy et al. 2012 (28)								Poor
Roghani et al. 2013 (26)								Poor

green: criterion met, yellow: criterion unclear, red: criterion not met
